# Serum metabolic signatures for Alzheimer’s Disease reveal alterations in amino acid composition: a validation study

**DOI:** 10.1007/s11306-023-02078-8

**Published:** 2024-01-05

**Authors:** Jonas Ellegaard Nielsen, Trygve Andreassen, Charlotte Held Gotfredsen, Dorte Aalund Olsen, Karsten Vestergaard, Jonna Skov Madsen, Søren Risom Kristensen, Shona Pedersen

**Affiliations:** 1 Department of Clinical Biochemistry, Aalborg University Hospital, Aalborg, Denmark; 2 Department of Biochemistry and Immunology, Lillebaelt Hospital, University Hospital of Southern Denmark, Vejle, Denmark; 3 Department of Circulation and Medical Imaging, Norwegian University of Science and Technology, Trondheim, Norway; 4 Central staff, St. Olavs Hospital HF, 7006 Trondheim, Norway; 5 Department of Chemistry, Technical University of Denmark, Kgs. Lyngby, Denmark; 6 Department of Regional Health Research, Faculty of Health Sciences, University of Southern Denmark, Odense, Denmark; 7 Department of Neurology, Aalborg University Hospital, Aalborg, Denmark; 8 Department of Clinical Medicine, Aalborg University, Aalborg, Denmark; 9 Department of Basic Medical Science, College of Medicine, Qatar University, QU Health, Doha, Qatar; 10 College of Medicine, Department of Basic Medical Science, Qatar University, 2713 Doha, Qatar

**Keywords:** Alzheimer, Metabolites, Biomarker, Serum, Nuclear magnetic resonance, Single molecule array

## Abstract

**Introduction:**

Alzheimer’s Disease (AD) is complex and novel approaches are urgently needed to aid in diagnosis. Blood is frequently used as a source for biomarkers; however, its complexity prevents proper detection. The analytical power of metabolomics, coupled with statistical tools, can assist in reducing this complexity.

**Objectives:**

Thus, we sought to validate a previously proposed panel of metabolic blood-based biomarkers for AD and expand our understanding of the pathological mechanisms involved in AD that are reflected in the blood.

**Methods:**

In the validation cohort serum and plasma were collected from 25 AD patients and 25 healthy controls. Serum was analysed for metabolites using nuclear magnetic resonance (NMR) spectroscopy, while plasma was tested for markers of neuronal damage and AD hallmark proteins using single molecule array (SIMOA).

**Results:**

The diagnostic performance of the metabolite biomarker panel was confirmed using sparse-partial least squares discriminant analysis (sPLS-DA) with an area under the curve (AUC) of 0.73 (95% confidence interval: 0.59–0.87). Pyruvic acid and valine were consistently reduced in the discovery and validation cohorts. Pathway analysis of significantly altered metabolites in the validation set revealed that they are involved in branched-chain amino acids (BCAAs) and energy metabolism (glycolysis and gluconeogenesis). Additionally, strong positive correlations were observed for valine and isoleucine between cerebrospinal fluid p-tau and t-tau.

**Conclusions:**

Our proposed panel of metabolites was successfully validated using a combined approach of NMR and sPLS-DA. It was discovered that cognitive-impairment-related metabolites belong to BCAAs and are involved in energy metabolism.

**Supplementary information:**

The online version contains supplementary material available at 10.1007/s11306-023-02078-8.

## Background

Neurodegenerative diseases, such as Alzheimer’s Disease (AD), account for a significant proportion of mortality, morbidity, and healthcare cost globally (Mattsson-Carlgren et al. 2020). Clinical examination alone is inadequate for guiding diagnosis, prognosis, and monitoring progress in research, clinical practice, and drug development. Imaging and biomarkers can aid diagnostics by providing an objective indicator of the underlying pathology. In the case of AD, this includes structural, functional, and molecular imaging, as well as measurements of signature proteins in the cerebrospinal fluid (CSF), i.e. amyloid-β (Aβ) and tau isoforms (Livingston et al. 2017; G. M. McKhann et al. 2011). In certain clinical situations, CSF levels of neurofilament light (Nf-L) protein, a marker for neuronal injury, have been used to diagnose neurodegenerative disorders (Khalil et al. 2018). However, with these current diagnostic methods, several drawbacks have to be accounted for, limiting their applicability as first-line screening tools. Although technological advances could increase the precision of these methods, their expense and lack of patient compliance prevent this from occurring. In addition, advanced scanning methods, including positron emission tomography, are expensive and less accessible for general practitioners (O’Brien and Herholz 2015), while CSF collection through a lumbar puncture is invasive (De Almeida et al. 2011). A blood sample may provide a matrix that could outweigh the drawbacks of the currently used biomarkers to diagnose patients with AD. With the benefits of blood being a biofluid in close contact with every organ in the body, its composition could reflect the potential state of the surrounding organ (Jacobs et al. 2005). The blood-brain barrier (BBB) separates the central nervous system (CNS) from the periphery, allowing only gaseous exchange, together with small ions, water- and small liposoluble-molecules to pass (Zlokovic 2011). However, during AD pathogenesis, the BBB becomes permeable (Baird et al. 2015), potentially enabling the identification of neuronal metabolites in blood samples.

Even though blood provides a non-invasive biological matrix for investigating disease pathology, its complexity impedes the findings of potential new biomarkers. The omics-era has aided in the realisation of the need to explore such complex samples, with metabolomics being one of the more recent members of the omics family (Hampel et al. 2016). Metabolomics covers the study of all metabolites in a cell, organ, or organism. Metabolites are small molecules < 1,500 Da and comprise amino acids, lipids, peptides, vitamins, etc. (Lamichhane et al. 2018), and are endpoints of the regulations at the genetic, transcript, and protein levels. Thus, small alterations of upstream molecules could substantially affect the concentration of a metabolite (J. Nielsen and Oliver 2005). Not only can disease progression cause metabolic perturbations, but environmental factors, treatments, and nutrition also play a role (Stringer et al. 2016). As for clinical applications, metabolic pathways have been shown to be evolutionarily conserved across species, thus bridging the gap between animal studies and human clinical trials (Wilkins and Trushina 2018). Nuclear magnetic resonance (NMR) spectroscopy is among the most informative techniques for studying metabolomics (Wishart 2008), and is able to efficiently analyse and detect hundreds of small molecules in a single measurement in human samples, including plasma and serum (Wang et al. 2013). Essentially, all metabolites present their own unique and reproducible NMR signature and thus can be used to explore metabolic processes and screen for the presence of known metabolites (Song et al. 2019). In addition, NMR is also non-destructive and more informative than other techniques, such as mass spectrometry; however, it lacks sensitivity and requires a larger quantity of sample material (Stringer et al. 2016; Wilkins and Trushina 2018).

Although biomarker studies contribute to the global search for a solution to the growing problem of the aging population, the literature demonstrates that replication efforts for many promising biomarker findings have failed (Voyle et al. 2015). Thus, the present study aimed to validate suggested metabolite biomarkers presented in our previous discovery study (J. E. Nielsen et al. 2021) and to provide additional information on metabolic perturbations associated with cognitive impairment. Using NMR-based metabolomics, the serum metabolic signatures from patients with mild to moderate AD were compared to those of cognitively healthy individuals. Furthermore, we validated our initial model using a larger validation cohort by incorporating the commonly identified metabolites and supplementing our findings with additional metabolic perturbations.

## Methods

### Study demographics

In our previous discovery study, 20 participants were enrolled, with 10 healthy controls and 10 patients with mild to modate AD. For this validation study, we increased the number of participants to 50, with 25 in each group either diagnosed with mild to moderate AD or as healthy controls. All subjects were Caucasian. The patients were recruited from the Department of Neurology, Aalborg University Hospital. Recruitment was performed consecutively at the time of diagnosis for the patients and prior to starting their treatment regimen. The diagnosis was based on the following criteria; the International Classification of Diseases and Related Health Problems 10th Edition (ICD_10_) (WHO n.d.), and the National Institute of Neurological and Communicative Disorders and Stroke and the Alzheimer’s Disease and Related Disorders Association (NINCDS-ADRDA) (G. McKhann et al. 1984). Paraclinical measurements comprised of Mini-Mental State Examination (MMSE), Addenbrooke’s Cognitive Examination (ACE), Functional Activities Questionnaire (FAQ), as well as Aβ, phospho-tau (p-tau), and total-tau (t-tau) measured in CSF. The paraclinical measurements were included when deemed necessary due to diagnostic uncertainty. Age- and sex-related donors were recruited from the blood bank at Aalborg University Hospital to serve as a comparison group for AD patients. In Denmark, blood donors are healthy unpaid volunteers without any apparent illnesses. Inclusion criteria for blood donors were an age > 65 years old and completion of a standard blood donor questionnaire describing physical and mental health, such as experiencing memory impairment, fatigue, or chest pain. Prior to inclusion in the study, all participants signed a written consent form. The study was approved by the local North Denmark Region Committee on Health Research Ethics (N-20150010) and conducted according to the Declaration of Helsinki.

### Sample collection and processing

Blood samples were drawn from study participants and processed as described in a previous study (Ellegaard Nielsen et al. 2020). Briefly, blood was collected from the median cubital vein using a 21-gauge needle in 10 mL clot activator tubes (BD Vacutainer, UK) and also 4 mL Ethylenediaminetetraacetic acid (EDTA) tubes (Vacuette, Greiner Bio-One, Austria). After sample collection, the blood was centrifuged twice at 2500 × *g* for 15 min at room temperature to obtain serum and plasma. After each centrifugation step, serum and plasma samples were aspirated to approximately 1 cm above the buffy coat or pellet. Finally, serum and plasma samples were aliquoted, snap-frozen using liquid nitrogen, and stored at − 80 °C until further analyses.

### Routine analyses

Organ markers were routinely measured in serum samples to ensure that none of the study participants had co-morbidities. The clinical biochemistry markers measured were alanine transaminase, albumin, carbamide, cholesterol, creatinine, C-reactive protein, glucose, high and low-density lipoprotein (HDL and LDL, respectively), lactate dehydrogenase (LDH), and triglycerides using the Alinity ci-series (Abbott, Chicago, IL, USA) and haemoglobin using either XN-9000 (Sysmex Europe SE, Germany) or Hb 201 DM (Hemocue AB, Sweden).

### Single Molecule Array

Aβ_40_, Aβ_42_, glial fibrillary acidic protein (GFAP), Nf-L, and p-tau181 were measured in EDTA plasma using the respective commercially available kits; Neurology 4-Plex E and P-Tau181 (Quanterix©, Billerica, MA, USA) by Single Molecule Array (SIMOA®) HD-X Analyzer. The analyses were performed according to the manufacturer’s instructions. In addition, the manufacturer’s commercial controls were applied for quality control.

### Nuclear magnetic resonance spectroscopy

Serum samples were initially thawed for one hour and then carefully diluted 1:1 dilution with sodium phosphate buffer (0.075 M, pH 7.4, 20% D_2_O in H_2_O, 6 mM NaN_3_, 4.6 mM 3-(trimethylsilyl)-2,2,3,3-tetradeuteropropanoic acid (TSP-d4)) and aliquoted into 5 mm NMR tubes. NMR spectra were recorded using a Bruker Avance Neo 600 MHz spectrometer attached to a BBI probe (Bruker Biospin GmbH, Rheinstetten, Germany). IconNMR (Topspin 4.1.1, Bruker Biospin GmbH, Rheinstetten, Germany) and Samplejet autosampler (Bruker Biospin GmbH, Rheinstetten, Germany) were used for sample handling and data acquisition. One-dimensional nuclear Overhauser effect (1D-NOESY) spectra, together with Carr-Purcell-Meiboom-Gill (CPMG), were recorded at 310 K using parameters for acquisition from Dona *et al.* (Dona et al. 2014). For the 1D-NOESY spectra, 96k data points were recorded, with 30 ppm spectral width. In contrast, CPMG spectra were recorded with 72k data points and a spectral width of 20 ppm. For both experiments, 32 scans with water suppression (25 Hz) during relaxation delay (4 s) and mixing time (NOESY, 10 ms) were used for recording. Free induction decays were Fourier transformed after artificial zero fillings up to 128k data points and 0.3 Hz line broadening. The spectra were automatically zero-order phase corrected. In accordance with the manufacturer, B.I.Methods (Bruker Biospin GmbH, Rheinstetten, Germany), reference samples were routinely measured and processed in automation for temperature calibration, water suppression determination, and external quantitative referencing. B.I.Quant-PS™ 2.0 (Bruker Biospin GmbH, Rheinstetten, Germany) was used to automatically quantify metabolites. A labeled spectrum with expanded regions for peak intensity comparison can be found in Supplementary file 1.

### Data analysis

For the validation cohort, 40 metabolites were identified using NMR. Information from the discovery cohort can be found in the discovery study (J. E. Nielsen et al. 2021). Metabolites were filtered for ≥ 70% valid values in at least one group before statistical analyses were conducted. Prior to statistical analysis, metabolites from the discovery- and validation-cohorts were adjusted for age and sex using a linear model. Also, prior to validation of the initial metabolic signature using multivariate data analysis, data were auto-scaled and mean-centered for metabolites common in both the discovery and validation cohorts.

Three models; Random Forest, Extreme Gradient Boosting (XGBoost), and sparse-partial least squared discriminant analysis (sPLS-DA), were tested and estimated by their performance using the following parameters; Area under the curve (AUC) and 95% confidence interval (CI) were reported to indicate the sensitivity and specificity of the model, together with the accuracy, positive predictive value (PPV), negative predictive value (NPV), and selected number of important metabolites. The Random Forest model performance was estimated by the out-of-bag error rate and optimal number of features was selected using the *randomForest* v4.7-1.1 and *Boruta* v8.0.0 R packages. XGBoosting was performed using the R package *xgboost* v1.7.5.1, with performance estimated by root mean squared error (RMSE). Optimal number of features were ranked according to importance score, and selected if importance score was > 0.1. As previously described (J. E. Nielsen et al. 2021), the sPLS-DA model was build with a 5-fold cross-validation (CV) repeated 100 times using the *mixOmics* v6.20.0 R package. The optimal number of selected features was estimated using the classification error rate. For visual purpose scores plot for sample groupings, loadings plot for weighted importance of selected metabolites, and receiver operating characteristic (ROC) curve are presented for the most optimal model.

Data were assessed for normality by Shapiro-Wilk test and histograms, and compared between the groups using a Student’s *t*-test, presented as mean ± standard deviations (SD). Correction for multiple comparison was also provided using the Benjamini-Hochberg false-discovery rate (FDR) corrected *p*-value. Nf-L and GFAP were corrected for age using a linear model (Vågberg et al. 2015). A significance level of *p* < 0.05 was chosen. Fold changes (FC) between groups were also calculated for the metabolites, using FC = (Metabolite_AD_ – Metabolite_Con_) / Metabolite_Con_. Correlations between important metabolites, selected by multivariate data analysis, and clinical data were investigated using Pearson’s ρ, with only the significant correlations presented. Data analysis and graphical representations were conducted using R version 4.2.2.

Network analysis was performed using the MetScape (version 3.1.3) App in CytoScape (version 3.9.1). The network was based on KEGG IDs from significantly altered metabolites between AD patients and healthy individuals. Raw NMR data for the validation cohort, clinical data, and input data for the network analysis can be found in Supplementary files 2, 3, and 4, respectively.

## Results

### Characteristics of study participants

The biochemical parameters, clinical test results for cognitive performances, corresponding clinical parameters, and SIMOA measurements for both study groups have been summarised in Table 1. Briefly, the majority of the biochemical measurements were within the standard reference intervals. A few, but significant differences were also observed between the AD patients and cognitively healthy individuals, including a slightly higher age (*p* = 0.00001), higher LDH levels (*p* = 0.03), and lower glucose levels in the AD patient group (*p* = 0.01). Patients who required additional cognitive testing and paraclinical measurements were identified, where AD patients presented with low MMSE (20.0 ± 4.5) and ACE (58.0 ± 16.5) scores and a high FAQ (11.8 ± 6.2) score, whereas paraclinical tests demonstrated elevated levels of CSF tau, p-tau (81.7 ± 25.0 ng/L) and t-tau (520.4 ± 102.4 ng/L), and decreased levels of CSF Aβ (682.8 ± 216.3 ng/L) for some of the patients, indicating extracellular tau accumulation and intracellular Aβ build-up. Plasma measurements of markers for neuronal injury and AD hallmark proteins were included as additional clinical information. Generally, AD patients had significantly higher plasma levels of Aβ_40_ (*p* = 0.002), GFAP (*p* = 0.01), Nf–L (*p* = 0.04), and p-tau181 (*p* = 0.00005) than healthy individuals; however, Aβ_42_ and Aβ_42_/Aβ_40_ ratio did not differ between the two groups (*p* = 0.5) and (*p* = 0.06), respectively. For GFAP and Nf-L unadjusted values for mean and SD are shown in Table 1.

**Table 1:** Characteristics of study participants

	Units	Con (*n* = 25)	AD (*n* = 25)	*p*-value	Reference interval
		Mean ± SD	Mean ± SD		
**Demographics**					
Age	Years	66.6 ± 1.3	75.7 ± 8.2	0.00001	–
Male/female	*n*	16 / 9	15 / 10	–	–
Ethnicity	*–*	Caucasian	Caucasian	–	–
**Biochemical measurements**					
ALAT	U/L	26.3 ± 8.6	22.3 ± 11.6	0.17	10.0–50.0
Albumin	g/L	41.0 ± 1.9	41.5 ± 1.9	0.37	34–45
Carbamide	mmol/L	5.8 ± 1.3	5.7 ± 1.5	0.77	3.1–8.1
Cholesterol	mmol/L	5.4 ± 0.9	5.5 ± 1.1	0.88	4.2–8.5
Creatinine	µmol/L	79.0 ± 10.2	83.4 ± 14.5	0.22	45–105
CRP	mg/L	1.9 ± 1.4	2.2 ± 2.9	0.57	< 8
Glucose	mmol/L	6.4 ± 1.7	5.4 ± 0.9	0.01	4.2–7.8
Haemoglobin	mmol/L	8.8 ± 0.7	8.5 ± 1.0 (*n* = 15)	0.45	7.3–10.5
HDL	mmol/L	1.5 ± 0.3	1.6 ± 0.4	0.35	0.7–1.9
LDL	mmol/L	3.2 ± 0.8	3.3 ± 0.9	0.71	2.2–5.7
LDH	U/L	170.2 ± 31.2	192.1 ± 38.7	0.03	105–255
Triglycerides	mmol/L	1.5 ± 0.8	1.3 ± 0.8	0.34	0.6–3.9
**Clinical parameters**					
MMSE	–	–	20.0 ± 4.5	–	–
ACE	–	–	58.0 ± 16.5 (*n* = 21)	–	–
FAQ	–	–	11.8 ± 6.2 (*n* = 21)	–	–
CSF Aβ	ng/L	–	682.8 ± 216.3 (*n* = 9)	–	> 500
CSF p-tau	ng/L	-	81.7 ± 25.0 (*n* = 9)	–	< 61
CSF t-tau	ng/L	–	520.4 ± 102.4 (*n* = 9)	–	< 450
**SIMOA**					
Aβ_40_	pg/mL	95.1 ± 10.2	108.7 ± 17.4	0.002	–
Aβ_42_	pg/mL	5.3 ± 1.0	5.6 ± 1.3	0.5	–
Aβ_42_/Aβ_40_	–	0.06 ± 0.009	0.05 ± 0.009	0.06	–
GFAP	pg/mL	88.6 ± 32.8	247.1 ± 277.9	0.01	–
Nf-L	pg/mL	12.5 ± 4.4	36.9 ± 24.5	0.04	–
p-tau181	pg/mL	1.8 ± 0.8	3.1 ± 1.3	0.00005	–

Demographics data of study participants together with biochemical measurements, cognitive test results, paraclinical measurements, and SIMOA measurements. Abbreviations; Aβ – Amyloid-β, ACE – Addenbrooke’s Cognitive Examination, AD – Alzheimer’s Disease, ALAT – Alanine transaminase, p-tau – Phosphorylated tau, CRP – C-reactive protein, CSF – Cerebrospinal fluid, FAQ – Functional Activities Questionnaire, GFAP – Glial fibrillary acidic protein, HDL – High-density lipoprotein, LDH – Lactate dehydrogenase, LDL – Low-density protein, MMSE – Mini-Mental State Examination, Nf-L – Neurofilament light, SD – Standard deviation, SIMOA – Single molecule array, t-tau – Total tau.

### Validation of metabolic signatures for Alzheimer’s Disease diagnostics

To validate the metabolic signature identified in our discovery study, NMR spectroscopy was applied to measure the concentration of serum metabolites in our validation study cohort. Three prediction models were tested for their performance based on AUC, accuracy, PPV, and NPV. These models included sPLS-DA, random forest, and XGBoost (Supplementary file 5). Based on these criteria, sPLS-DA showed the highest performance with five selected metabolites building the model (pyruvic acid, valine, histidine, isoleucine, and creatine), while random forest performed the second best with four selected metabolites (histidine, valine, pyruvic acid, and creatine), and lastly the XGBoost with four metabolites (histidine, pyruvic acid, valine, and tyrosine). Thus, sPLS-DA was selected as our data's most optimal validation model.

**Fig. 1:**
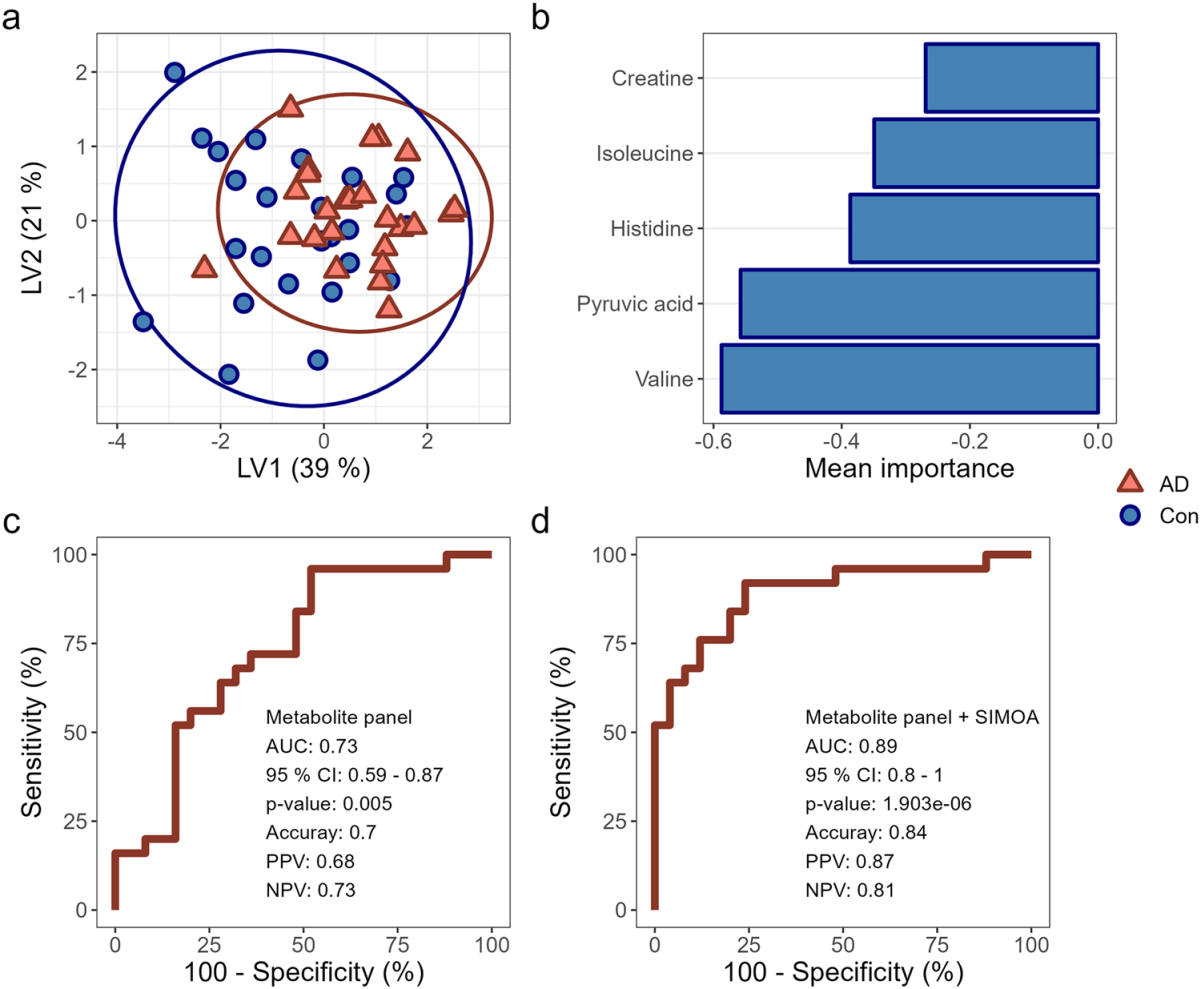
Validation of metabolic signature for cognitive impairment. **a** Scores plot for sparse-partial least squared discriminant analysis (sPLS-DA), with each score representing a sample. **b** Loadings plot for selected metabolites representing their mean importance for sample grouping reflected in the scores plot. The colour-coding of the bars indicates their importance for the corresponding group. **c** Receiver operating characteristics (ROC) curve indicates the ability of the model to distinguish the groups. Together with the ROC curve is the area under the curve (AUC) with the presented 95% CI, accuracy, PPV, and NPV. **d** ROC curve of selected metabolites combined with significantly altered markers of neurodegeneration (Aβ_40_, GFAP, Nf-L, p-tau181) showing an improved diagnostic efficacy, also presented with AUC and the 95% CI, accuracy, PPV, and NPV. Abbreviations; AD – Alzheimer’s Disease, AUC – Area under the curve, CI – Confidence interval, Con – Healthy controls, LV – Latent variable, NPV – Negative predictive value, PPV – Positive predictive value.

The validation model showed a small overlap between the patient and control groups, as seen in the scores plot of the measured serum samples (Fig. 1a). Based on the validated model, five metabolites significantly contribute to sample grouping, accounting for 39% of the group variation (Fig. 1b). Consequently, the model had an AUC performance of 0.73 (95% CI = 0.59–0.87) for discriminating AD patients from cognitively healthy individuals (Fig. 1c). Furthermore, the model had an accuracy = 0.70, PPV = 0.68, and NPV = 0.73, indicating its diagnostic value. Interestingly, when adding the significantly altered proteins (Aβ_40_, GFAP, Nf-L, and p-tau181) to the validation model, improved its diagnostic performance, resulting in an AUC of 0.89 (95% CI = 0.80–1.00) with an accuracy of 0.84, PPV of 0.87, and NPV of 0.81 (Fig. 1d).

**Fig. 2:**
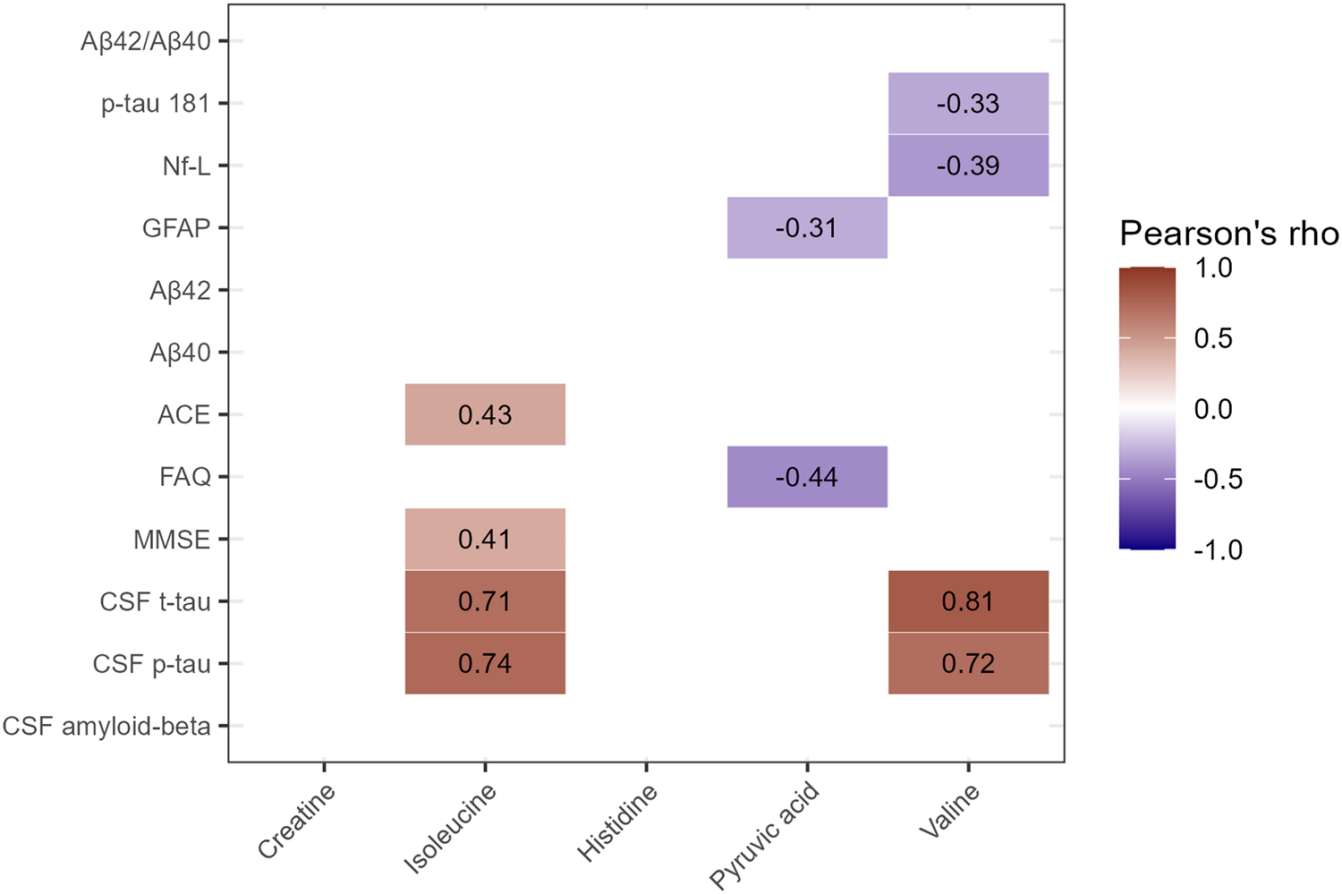
Correlogram of metabolites of interest and clinical parameters. Only correlations shown to be significant are included. The colour of the square indicates if the correlation is positive (red) or negative (blue), and the intensity of the colour corresponds to the level of the correlation. Abbreviations; ACE – Addenbrooke’s cognitive examination, CSF – Cerebrospinal fluid, FAQ – Functional activities questionnaire, GFAP – Glial fibrillary acidic protein, MMSE – Mini-mental state examination, Nf-L – Neurofilament light, p-tau – Phospho-tau, t-tau – Total-tau.

Furthermore, the selected panel of five metabolites was correlated against the clinical data and markers of neuronal damage to determine their possible association with neurodegenerative diseases (Fig. 2). Especially isoleucine and valine exhibited a strong significant positive correlation with CSF levels of p-tau and t-tau with a Pearson’s correlation of 0.74 and 0.71 for isoleucine and 0.72 and 0.81 for valine, respectively. Pyruvic acid showed a moderate negative correlation with cognitive scoring test FAQ (ρ = − 0.44), while isoleucine showed moderate positive correlations with cognitive scoring tests MMSE (ρ = 0.41) and ACE (ρ = 0.43).

Two of the metabolites measured in both the discovery and validation data sets were significantly altered when comparing healthy and diseased individuals (Table 2). These two metabolites exhibited similar changes in both the discovery and validation studies. Furthermore, tyrosine and leucine also presented as significantly changed in the validation cohort, however, not in the discovery cohort, while histidine presented as significantly changed only in the discovery cohort. The unadjusted mean ± SD values are shown in Table 2.

**Table 2:** Common significantly altered metabolites

Metabolite [mmol/L]	Con		AD		FC	*p*-value	FDR
	Mean	SD	Mean	SD			
Discovery study							
Valine	0.118	0.019	0.092	0.011	− 0.2	0.007	0.07
Histidine	0.037	0.002	0.032	0.002	− 0.1	0.009	0.07
Pyruvic acid	0.032	0.007	0.026	0.004	− 0.2	0.03	0.19
Validation study							
Tyrosine	0.071	0.016	0.051	0.011	− 0.3	0.006	0.09
Valine	0.275	0.052	0.214	0.040	− 0.2	0.01	0.09
Pyruvic acid	0.118	0.031	0.079	0.027	− 0.3	0.02	0.09
Leucine	0.111	0.035	0.078	0.017	− 0.3	0.02	0.09

Two metabolites were dysregulated in serum samples between cognitively affected and healthy individuals in both the discovery and validation studies, sorted according to *p*-value. Abbreviations; AD – Alzheimer’s Disease, Con – Healthy controls, FC – Fold change, SD – Standard deviation

### Metabolic alterations in the validation cohort

To extrapolate novel metabolic information, serum samples from the validation study were examined for significantly altered metabolites between the groups. This brought the total number of significantly different metabolites between the groups to five. Four of these metabolites were previously identified as significant in the discovery cohort, only valine was previously reported as significantly altered (Table 3). The unadjusted mean ± SD values are shown in Table 3. Significance testing of unadjusted metabolites can be found in Supplementary file 6. Potential significant differences due to age could be removed by adjustment, however, this difference could also be due to the AD patients on average being older or having different metabolite concentrations due to the disease state, thereby eliminating this relevant difference.

**Table 3:** Significantly altered metabolites in the validation cohort

Metabolite (ppm, multiplicity) [mmol/L]	Con		AD		FC	*p*-value	FDR
	Mean	SD	Mean	SD			
Tyrosine (7.19, app. d)	0.071	0.016	0.051	0.011	− 0.3	0.006	0.13
Valine (1.02, d)	0.275	0.052	0.214	0.040	− 0.2	0.01	0.13
Lysine (3.03, t)	0.220	0.043	0.175	0.036	− 0.2	0.02	0.13
Pyruvic acid (2.36 s)	0.118	0.031	0.079	0.027	− 0.3	0.02	0.13
Leucine (0.94(s) & 0.96(d))	0.111	0.035	0.078	0.017	− 0.3	0.02	0.14

Significantly altered metabolites measured in serum samples comparing cognitively affected with healthy individuals, sorted according to the *p*-value. Abbreviations; AD – Alzheimer’s Disease, Con – Healthy controls, FC – Fold change, FDR – False-discovery rate, SD – Standard deviation.

**Fig. 3:**
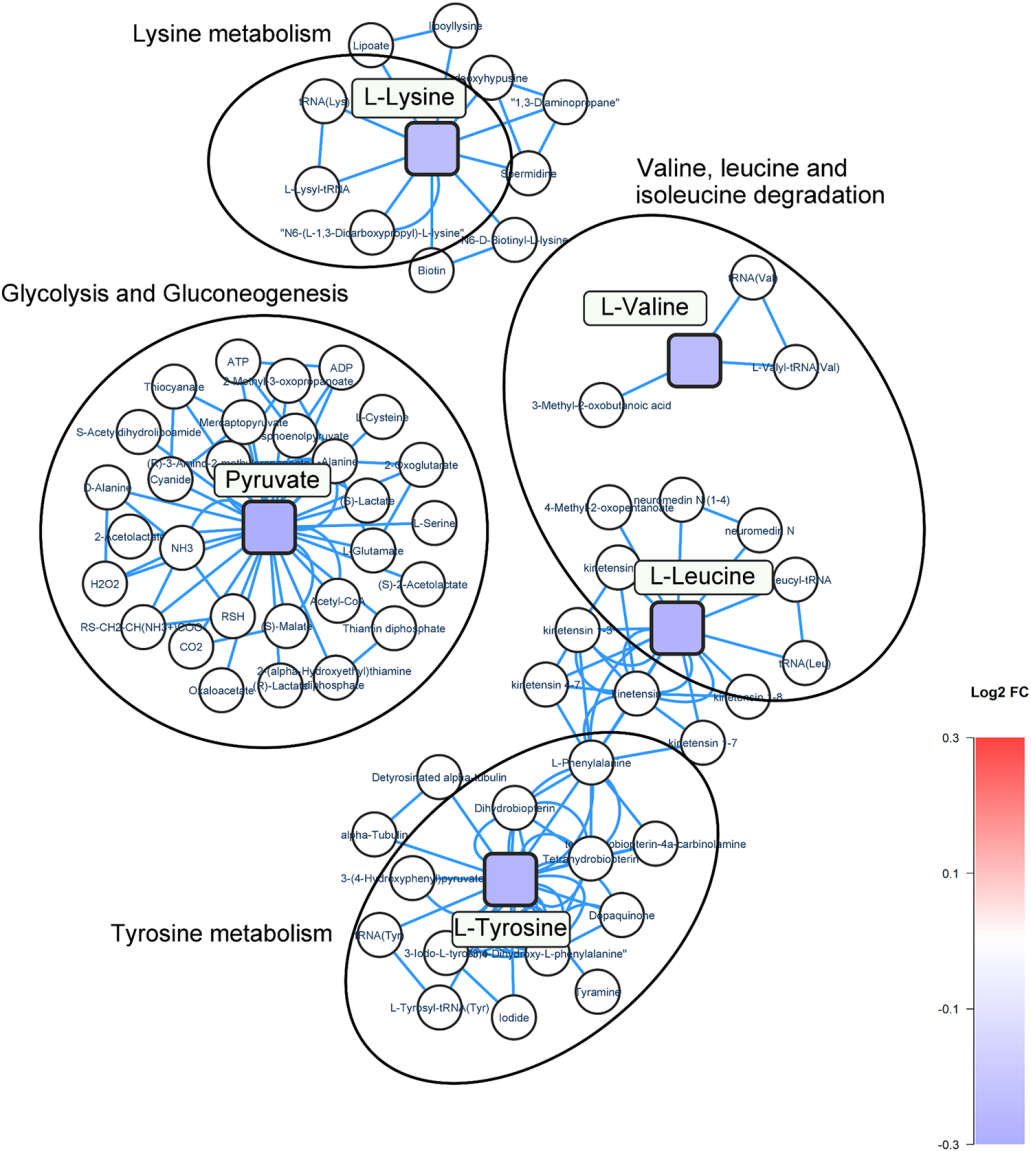
Network analysis of dysregulated pathways related to cognitive impairment. Square nodes represent altered metabolites identified in the validation study, and circular nodes represent metabolites as part of the pathways not identified in the study. Colour of the square nodes represents the log_2_ FC value of the corresponding metabolite, according to alterations between the groups, with blue indicating a downregulation, and red indicating an upregulation. Metabolites are mapped according to their KEGG IDs

A network analysis was performed to investigate biological pathways for the significantly altered metabolites related to cognitive impairment (Fig. 3). Pyruvate involved in glycolysis and gluconeogenesis (log_2_ FC = − 0.33, *p*-value = 0.02) was the most reduced metabolite in relation to AD. In addition, the metabolic pathways of Lysine, Tyrosine, and branch-chained amino acids (BCAAs); valine and leucine, were also modified. This validation study identified and confirmed changes to BCAA metabolism previously found in the discovery study; leucine (log_2_ FC = 0.3, *p*-value = 0.02) and valine (log_2_ FC = − 0.2, *p*-value = 0.01).

## Discussion

In this study, we examined serum-derived metabolites associated with cognitive impairment in patients with mild to moderate AD compared to healthy individuals. The primary objective was to validate the significance of a panel of metabolites previously identified in the discovery study (J. E. Nielsen et al. 2021). The secondary objective was to add novel information not previously identified.

As previously mentioned, reproducibility is one of the more significant obstacles in biomarker studies (Mattsson-Carlgren et al. 2020). The authors of the referred study raised several crucial points to improve the reproducibility of future biomarker studies for neurodegenerative disease. These aspects range from cohort-related factors to independent validation. In the presented study, we have sought to comply with these recommendations, including; 1) consecutively recruitment of study participants to enroll more heterogenous groups, such that multiple factors attenuating the effect of the biomarker to avoid overestimation, 2) avoiding confounding factors, such as the presence of co-morbidities affecting measured biomarker levels, 3) performing validation in a separate validation cohort, as internal CV, i.e. when splitting a single cohort, has shown to fail when replicating the models in independent cohorts (Burnham et al. 2014; Voyle et al. 2015), possibly due to systematic bias between the groups, 4) reporting not only the overall measure of performance, such as the AUC but also including the parameters PPV, NPV, etc., and lastly 5) metabolomics was performed in a different lab in the validation study compared to the discovery study, thereby also accounting for between-lab variability. (Mattsson-Carlgren et al. 2020)

We examined the serum metabolome of our study participants through NMR spectroscopy in combination with multivariate data analysis. Three models were tested using the discovery and validation data sets. Overall, these models primarily selected the same metabolites, indicating the relevance of these metabolites for distinguishing cognitively impaired persons from healthy individuals. The commonly selected metabolites included pyruvic acid, valine, and histidine. Pyruvic acid, or pyruvate, is the end-product of glycolysis and the substrate for mitochondrial adenosine triphosphate (ATP) synthesis. The nervous system is vulnerable to alterations in pyruvate metabolism due to the high ATP demand (Gray et al. 2014), which is used to maintain neuronal activity and homeostasis of the extracellular space and to defend against oxidative stress (Bak et al. 2006; García-Nogales et al. 2003). In contrast, to our findings, increased levels of pyruvate have been observed in CSF of AD patients (Parnetti et al. 1995), but similar alterations were identified in blood samples from patients with Parkinson’s disease (Ahmed et al. 2009). As stated in the discovery study, valine and histidine are well-studied amino acids with respect to AD pathology (J. E. Nielsen et al. 2021). Consequently, the results of the present study further validate their importance related to AD. In accordance with previous findings identifying decreased levels of valine in serum (Xiong et al. 2022) and CSF and positive correlations between CSF-valine and MMSE score, researchers have continued investigating this particular amino acid in relation to AD (Vignoli et al. 2020). Valine was identified as a potential marker for predicting the transition from mild cognitive impairment to AD (Xiong et al. 2022). Histidine is an essential amino acid (Kim and Kim 2020), and a known scavenger of hydroxyl radicals, part of the reactive oxygen species (Cai et al. 1995). Using a cell model for anti-aging effects, increased proliferation and neurogenesis, as well as up-regulation of anti-oxidant enzymes, have been demonstrated as positive effects of histidine (Kim and Kim 2020). As mentioned earlier, BBB breakdown occurs during AD pathogenesis with the potential presence of neuronal metabolites in the circulatory system. Thus, the metabolic shift between healthy and diseased individuals observed in this study could also be due to the presence of neuronal-derived metabolites entering the bloodstream in AD subjects, while not being present in healthy individuals due to an intact BBB.

As an additional clinical characteristic parameter, we included measurements of established non-disease specific markers of neurological damage (GFAP and Nf-L), as well as hallmark targets of AD (Aβ_40_, Aβ_42_, Aβ_42_/ Aβ_40_, and p-tau181) measured in blood, as the literature strongly implicates their diagnostic performance and significance in relation to neurological disease. (Chatterjee et al. 2022; Smirnov et al. 2022). Overall, the present study confirms previous findings; however, we found significantly elevated levels of Aβ_40_ and no difference in Aβ_42_ concentrations and Aβ_42_/Aβ_40_ ratios. This may be due to underlying cardiovascular conditions, such as hypertension, ischemic heart disease, and cardio-protective medications, which have been shown to influence plasma concentrations of Aβ. (Janelidze et al. 2016). However, this indifference could also be associated to the cohort size of the study needing more power to confirm a significant difference. In the biochemical measurements, glucose and LDH levels were significantly different between the control group and the patients, but both groups had levels within the normal range. Interestingly, reduced glucose utilization has been shown in AD brains (Kumar et al. 2022), and this could also explain the observed significantly lower pyruvate concentration. Although, blood donors are encouraged to have eaten prior to blood donation, which also could be the reason for the significance in blood glucose levels.

Even though our results confirmed important findings and contributed to the search for valid blood-based biomarkers to aid in diagnosing AD, it is essential to note the limitations of our study. First, although the patient group was clinically confirmed to have AD, not all patients underwent neuropsychological testing or had CSF proteins measured because their physician deemed it unnecessary for the patient's diagnosis. Secondly, to thoroughly verify our metabolite panel as a diagnostic tool for AD, it would be necessary to test its accuracy against other neurodegenerative diseases and different stages of AD. Thirdly, we discovered a significant age difference between our study groups, with AD patients being, on average, older than the control group. Unfortunately, it is not possible to recruit older blood donors, however, adjustment for age was performed on metabolite values prior to statistical analsysis. Fourthly, adding CSF samples to a study of the metabolome in relation to cognitive impairment can strengthen biomarker panels identified in the peripheral system. Lastly, both *p*-value and FDR corrected values were reported to minimize type II errors, however, multivariate statistics were also applied to encompass the overall information in the data, including covariance and correlation between metabolites.

Our findings validated the significance of the identified metabolite biomarker panel from the discovery study in distinguishing cognitively healthy individuals from patients with cognitive impairment. In addition, we evaluated various models to validate the performance of our panel, finding sPLS-DA to be the best fit. Lastly, new insights into disruptions in energy metabolism were uncovered.

## Conclusions

In the current study, we validated our blood-based biomarker model derived from a discovery study consisting of five metabolites; pyruvic acid, valine, histidine, isoleucine, and creatine. The novel information provided by the validation cohort confirmed the involvement of significantly altered metabolites in the BCAAs metabolism. Moreover, metabolic changes in the energy metabolism were observed. Combining the proposed metabolite biomarker panel with neurodegenerative markers in plasma increased the diagnostic value. Although our validation yielded very intriguing results, the diagnostic performance of this panel of metabolic markers must also be assessed against other types of neurodegenerative diseases and various stages of AD progression.

### Supplementary information

Below is the link to the electronic supplementary material.

Supplementary material 1 (PDF 57.7 kb)

Supplementary material 2 (XLSX 27.7 kb)

Supplementary material 3 (XLSX 20.5 kb)

Supplementary material 4 (XLSX 8.9 kb)

Supplementary material 5 (DOCX 12.8 kb)

Supplementary material 6 (DOCX 14.3 kb)

## Data Availability

All data generated or analysed during this study are included in this published article (and its supplementary information files).
