# Supplementary figures and images for: Serum metabolic signatures for Alzheimer’s Disease reveal alterations in amino acid composition: a validation study
Source: Metabolomics. 2024 Jan 5;20(1):12. doi: 10.1007/s11306-023-02078-8 (PMC10770204; doi:10.1007/s11306-023-02078-8)

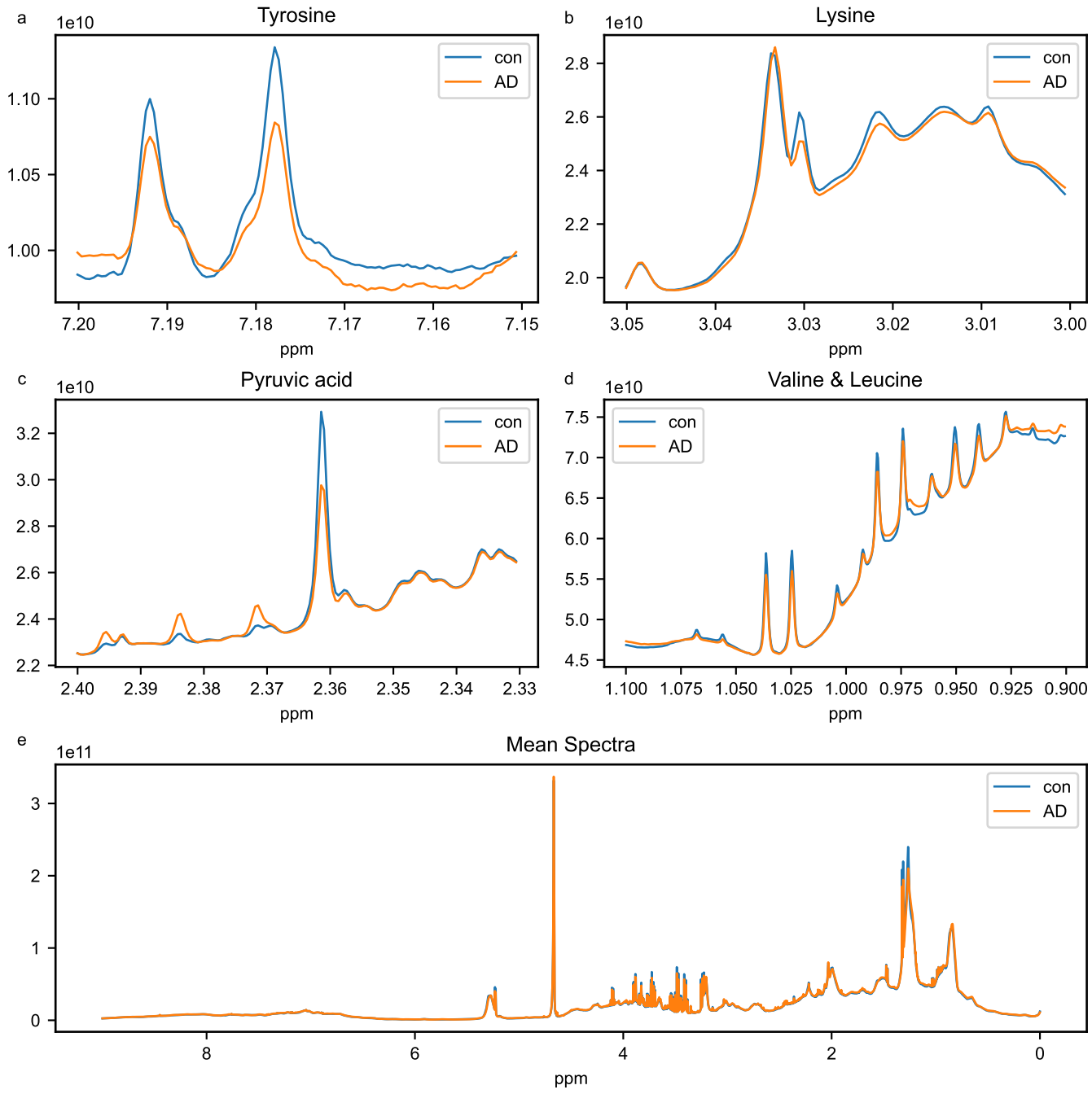

Supplement: Supplementary file 1 — Supplementary material 1 (PDF 57.7 kb) [file 11306_2023_2078_MOESM1_ESM.pdf]
