# Supplementary material for: Serum metabolic signatures for Alzheimer’s Disease reveal alterations in amino acid composition: a validation study
Source: Metabolomics. 2024 Jan 5;20(1):12. doi: 10.1007/s11306-023-02078-8 (PMC10770204; doi:10.1007/s11306-023-02078-8)
Supplement: Supplementary file 5 — Supplementary material 5 (DOCX 12.8 kb) [file 11306_2023_2078_MOESM5_ESM.docx]

**Supplementary Material File 4.** Performance of prediction models.

| **Model** | **AUC** | **95 % CI** | **Accuracy** | **PPV** | **NPV** | **Features** |
| --- | --- | --- | --- | --- | --- | --- |
| sPLS-DA | 0.89 | 0.79 – 0.98 | 0.86 | 0.88 | 0.85 | 5 |
| Random Forest | 0.87 | 0.77 – 0.97 | 0.76 | 0.84 | 0.71 | 4 |
| XGBoost | 0.84 | 0.73 – 0.95 | 0.74 | 0.72 | 0.76 | 3 |

Three validation models and their diagnostic performance; sparse-partial least squared discriminant analysis, random forest, and extreme gradient boosting. Abbreviations; AUC – Area under the curve, CI – Confidence interval, NPV – Negative predictive value, PPV – Positive predictive value, sPLS-DA – Sparse-partial least squared discriminant analysis, XGBoost – Extreme gradient boosting.
