# Supplementary material for: Serum metabolic signatures for Alzheimer’s Disease reveal alterations in amino acid composition: a validation study
Source: Metabolomics. 2024 Jan 5;20(1):12. doi: 10.1007/s11306-023-02078-8 (PMC10770204; doi:10.1007/s11306-023-02078-8)
Supplement: Supplementary file 6 — Supplementary material 6 (DOCX 14.3 kb) [file 11306_2023_2078_MOESM6_ESM.docx]

**Table S1.** Significantly altered metabolites in the validation cohort without adjustment.

| **Metabolite [mmol/L]** | **Con** | | **AD** | | **FC** | ***p*-value** | **FDR** |
| --- | --- | --- | --- | --- | --- | --- | --- |
|  | **Mean** | **SD** | **Mean** | **SD** |  |  |  |
| Tyrosine | 0.07 | 0.02 | 0.05 | 0.01 | -0.3 | 0.000006 | 0.0002 |
| Pyruvic acid | 0.12 | 0.03 | 0.08 | 0.03 | -0.3 | 0.00002 | 0.0002 |
| Valine | 0.27 | 0.05 | 0.21 | 0.04 | -0.2 | 0.00003 | 0.0003 |
| Leucine | 0.11 | 0.03 | 0.08 | 0.02 | -0.3 | 0.0001 | 0.001 |
| Lysine | 0.22 | 0.04 | 0.17 | 0.04 | -0.2 | 0.0002 | 0.001 |
| Histidine | 0.12 | 0.02 | 0.10 | 0.02 | -0.2 | 0.0006 | 0.003 |
| Glycerol | 0.19 | 0.05 | 0.28 | 0.11 | 0.5 | 0.001 | 0.007 |
| 3-Hydroxybutyric acid | 0.07 | 0.07 | 0.16 | 0.14 | 1.3 | 0.008 | 0.03 |
| Acetoacetic acid | 0.01 | 0.01 | 0.02 | 0.02 | 1.5 | 0.01 | 0.03 |
| Alanine | 0.50 | 0.07 | 0.44 | 0.09 | -0.1 | 0.01 | 0.03 |
| Glucose | 6.68 | 1.88 | 5.55 | 1.05 | -0.2 | 0.01 | 0.03 |
| Citric acid | 0.12 | 0.03 | 0.14 | 0.04 | 0.2 | 0.02 | 0.04 |
| Isoleucine | 0.07 | 0.03 | 0.05 | 0.02 | -0.2 | 0.02 | 0.04 |
| Succinic acid | 0.003 | 0.002 | 0.006 | 0.006 | 1.1 | 0.02 | 0.046 |
| Phenylalanine | 0.06 | 0.01 | 0.05 | 0.01 | -0.1 | 0.04 | 0.07 |
| Lactic acid | 2.26 | 0.54 | 1.96 | 0.52 | -0.1 | 0.049 | 0.1 |

Significantly altered metabolites measured in serum samples comparing cognitively affected with healthy individuals, sorted according to the *p*-value. Abbreviations; AD – Alzheimer’s Disease, Con – Healthy controls, FC – Fold change, FDR – False-discovery rate, SD – Standard deviation.
